# Personalising monitoring for chemotherapy patients through predicting deterioration in renal and hepatic function

**DOI:** 10.1002/cam4.6418

**Published:** 2023-08-23

**Authors:** Pinkie Chambers, Matthew Watson, John Bridgewater, Martin D. Forster, Rebecca Roylance, Rebecca Burgoyne, Sebastian Masento, Luke Steventon, James Harmsworth King, Nick Duncan, Noura al Moubayed

**Affiliations:** ^1^ UCL School of Pharmacy London UK; ^2^ Cancer Division University College London Hospitals NHS Foundation Trust London UK; ^3^ Department of Computer Science Durham University Durham UK; ^4^ UCL Cancer Institute London UK; ^5^ Evergreen Life Manchester UK; ^6^ University Hospitals Birmingham NHS Foundation Trust Birmingham UK

**Keywords:** chemotherapy, hepatic, machine learning, renal, treatment‐dose

## Abstract

**Background:**

In those receiving chemotherapy, renal and hepatic dysfunction can increase the risk of toxicity and should therefore be monitored. We aimed to develop a machine learning model to identify those patients that need closer monitoring, enabling a safer and more efficient service.

**Methods:**

We used retrospective data from a large academic hospital, for patients treated with chemotherapy for breast cancer, colorectal cancer and diffuse‐large B‐cell lymphoma, to train and validate a Multi‐Layer Perceptrons (MLP) model to predict the outcomes of unacceptable rises in bilirubin or creatinine. To assess the performance of the model, validation was performed using patient data from a separate, independent hospital using the same variables. Using this dataset, we evaluated the sensitivity and specificity of the model.

**Results:**

1214 patients in total were identified. The training set had almost perfect sensitivity and specificity of >0.95; the area under the curve (AUC) was 0.99 (95% CI 0.98–1.00) for creatinine and 0.97 (95% CI: 0.95–0.99) for bilirubin. The validation set had good sensitivity (creatinine: 0.60, 95% CI: 0.55–0.64, bilirubin: 0.54, 95% CI: 0.52–0.56), and specificity (creatinine 0.98, 95% CI: 0.96–0.99, bilirubin 0.90, 95% CI: 0.87–0.94) and area under the curve (creatinine: 0.76, 95% CI: 0.70, 0.82, bilirubin 0.72, 95% CI: 0.68–0.76).

**Conclusions:**

We have demonstrated that a MLP model can be used to reduce the number of blood tests required for some patients at low risk of organ dysfunction, whilst improving safety for others at high risk.

## Graphical abstract

We have found that less than 15% of patients suffer deterioration in renal and hepatic function whilst receiving chemotherapy. Our prediction model supports stratification of organ function monitoring has clinical utility for those that are high risk; guiding additional monitoring and safety netting. For those patients at low risk, our model would support a reduction in blood tests. This work has potential to save many blood tests and hospital encounters for patients.

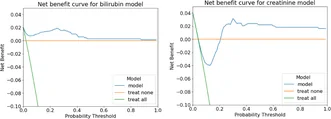

## 1 INTRODUCTION

Despite advances in targeted and immune therapies, cytotoxic chemotherapy remains the gold standard, first line treatment for many common cancers including breast cancer, colorectal cancer and diffuse large B‐cell lymphoma (DLBCL).^1^ These anticancer agents are characterised by a narrow therapeutic index and large interindividual pharmacokinetic variability. This means that small changes in plasma concentration, consequent to organ function impairment, may lead to unacceptable toxicity.^2^ A systematic review published by Krens and colleagues^3^ suggested dose adjustments when initiating chemotherapy treatment for patients with pre‐existing renal or hepatic impairment. This guidance did not, however, advise on subsequent cycle dosing. Furthermore, clinical evidence for the guidance was modest.

Routine clinical practice is to assess kidney and liver function prior to each treatment cycle^4^; however, the value of this process with respect to detecting significant changes that necessitate chemotherapy dose modifications is uncertain. Where patients are very unlikely to experience significant changes in renal and hepatic function during chemotherapy, monitoring at every cycle may be unnecessary, and for many patients involves increased waiting times and unnecessary blood tests.^5^ With the use of chemotherapy increasing,^6^ there is a need to rationalise the amount of tests that are conducted for patients. Accurate stratification of patients, in order to conduct blood tests only for those that are likely to experience deterioration in renal and hepatic function during chemotherapy, would have benefits both in terms of patient experience and reducing the cost of delivering care.

There is an opportunity to leverage machine learning (ML) to support this stratification of patients into high‐ and low‐risk groups. In the United Kingdom, all hospitals use Electronic Prescribing (EP) systems to prescribe cancer treatments. These systems used for prescribing contain information of drugs received, dosing and also demographic information, laboratory parameters and details of concurrent medication, providing comprehensive data for use in model development.^7^ Developed models could be employed to guide clinicians in providing individualised blood testing schedules for patients treated with chemotherapy, enabling safer treatment for those that are likely to suffer deterioration, and reduced tests for those unlikely to encounter changes. We therefore aimed to develop a model to predict the risk of an individual patient experiencing grade changes for creatinine, a marker of kidney function, and bilirubin, a marker of liver function.

## 2 METHODS

This was a retrospective data study to develop and validate a multilayer perceptrons (MLPs) model, and is reported using the Transparent Reporting of a multivariable prediction model for Individual Prognosis Or Diagnosis' (TRIPOD) statement,^8^ a 22‐item checklist that guides the reporting of the design, conduct, analysis and interpretation of prediction modelling studies.^9^ We chose to develop a MLP model rather than a prognostic multivariable logistic regression model^10^ due to the multiple levels within our dataset, limiting the performance of statistical models.^11^ MLPs have been shown to outperform multivariable regression models in similar applications^12^ and so were chosen in order to maximise the performance of the model. Additionally, recent developments in the field of explainable machine learning would allow for clinical interpretation.

Data from one hospital were used in the development of the model, and another hospital to validate the model. Both hospitals were specialist cancer hospitals located in the United Kingdom.

### 2.1 Inclusion and exclusion criteria

Patients' records were included if they were aged 18 or over. Patients were identified through the chemotherapy EP system at each site, and all data were extracted for the period 01 January 2013–31 December 2018. We used the first chemotherapy treatment date derived from the EP data as the index date for entry to the cohort during the study period. Patients were then followed up until the administration of the sixth cycle of treatment. It should be noted that the sixth cycle was not necessarily the final chemotherapy cycle administered to the patient, but this timepoint was used for the end of follow‐up period in this study.

Data were restricted to the following three tumour groups: breast, colorectal and diffuse large B‐Cell lymphoma, identified using the ICD10 codes^13^ C50, C83, C19, C19, C20 and C21. In the case of breast cancer, we included only those patients with early‐stage breast cancer (stages 1–3). For colorectal cancers, we included all patients receiving their first treatments for any stage disease. Patients were only included if they received first‐line treatment with the following regimens: epirubicin and cyclophosphamide (EC) only, or in combination with fluorouracil (FEC); docetaxel alone or with cyclophosphamide; irinotecan modified de Gramont (IRMDG); oxaliplatin modified de Gramont (FOLFOX); oxaliplatin and capecitabine (OXCAP); and rituximab, cyclophosphamide, vincristine and prednisolone (RCHOP).

Patients were excluded from the dataset if they received only one cycle of treatment. Additionally, patients were excluded where the second cycle of treatment was administered beyond a period of 60 days from the date of first treatment.

### 2.2 Analysis

#### 2.2.1 Data variables

The outcome of interest was any deterioration in kidney or liver function. This was defined by grade change in creatinine or bilirubin among patients included, at any cycle following cycle two. We chose bilirubin as an outcome measure rather than alanine aminotransferase (ALT), as increases in this marker are common with a number of chemotherapeutic agents and rarely cause clinical concern or reflect dysfunction of the liver.^14^, ^15^

The presence of grade changes was determined using the Common Terminology Criteria for Adverse Events (CTCAE) guidelines; however, we used a modified CTCAE toxicity boundary that reduced abnormal limits to reduce false negatives, which would inherently cause an increase in false positives (this adjustment is detailed in the Appendix S1). Post‐cycle two was used as the outcome, as it is understood that many toxicities to treatment occur during the first cycle of chemotherapy. Additionally, it is believed that it would not be clinically acceptable to remove any blood test monitoring at cycle 2.

The predictors incorporated into the development model are routinely recorded in EP systems: baseline, cycle 1 and cycle 2 blood results, patient demographics, comorbidities and details of treatment, including the proportion of doses received compared to the calculated standard dose. The same collection of blood results was used across all cycles: Absolute neutrophil count (ANC), haemoglobin level (HB), creatinine level, alanine aminotransferase (ALT) level and bilirubin level. These laboratory variables are standardly available for the majority of patients commencing chemotherapy, and have been identified as important predictors in other studies where toxicity outcomes have been assessed.^16^, ^17^ Demographic information included patient's age on commencing treatment, gender, cancer type, ethnicity, height and weight. Treatment information used were regimen received and relative dose intensity.

Day 1 (the date of the start of the 1st cycle) was used as the index date, and each blood test date was ordered in relation to number of days from the index date. Baseline results were any results that either preceded the index date by 7 days, or were taken within 72 hours following the index date. If there was more than one baseline value available, we used the value closest to the index date.

Drug regimens included were categorised by cycle length, a standard time interval for a particular regimen. By using the standard cycle length of either 14 or 21 days, we were able to determine if treatment administration had been delayed.

#### 2.2.2 Missing data

We made the decision to exclude any patient with missing values at cycles 1, 2 and 3 and report the numbers excluded.

#### 2.2.3 Machine learning models

Data from Hospital 1 were used as a training cohort, to predict deterioration of one grade or greater in creatinine and bilirubin following cycle 2 of chemotherapy. In deep learning, a set of training data is passed through multiple ‘layers’ of a model; these layers are composed of simple non‐linear operations with the representation produced by one layer being fed into the next layer, which in turn transforms the data into an abstract representation.^18^

We used multilayer perceptrons (MLPs) as the model of choice. MLPs consist of an input layer (the predictors) followed by one or more fully‐connected ‘hidden’ layers (‘neurons’) that are composed of regressions.^19^ The training data were randomly split to perform an internal validation to reduce any overfitting. We used an 80%, 10%, 10% split for the training, test and validation, respectively, using stratified sampling to ensure each set was representative of the whole population. All data were first normalised to ensure that the magnitude of each feature did not affect the MLPs outcome. Ten‐fold cross validation was utilised to evaluate the performance of the trained models. This technique provided a less‐biased, less‐optimistic evaluation of a model^20^ than just using a simple train/test dataset split.

The output of the trained models was the patient's predicted creatinine/bilirubin value. These predicted values were then used to place patients into one of two groups: the patient is predicted a grade change (as defined by the CTCAE guidance), or the patient's bilirubin/creatinine grade is predicted to remain stable. These groups are defined in Table S1, with the boundaries for each group being derived from CTCAE guidelines.^3^ Importantly, the grade boundaries defined were set slightly lower than those in the original CTCAE guidance. This was by design to ensure that any patients with predicted creatinine/bilirubin values close to the CTCAE grade boundaries would be classified as potentially experiencing a grade change. This would purposefully increase the number of false positive (FP) classifications, and reduce the number of false negatives (FN). From a clinical perspective, a larger number of FPs is preferable to a large number of FNs, as any FP will still receive blood tests at every cycle whereas a FN patient will not, thereby provide safety‐netting.

As part of the training process, several sets of hyperparameters were chosen—these are values that optimise the way in which the MLP models are trained. For example, the number of layers in the MLPs must be selected pretraining, as must the size of these layers. Hyperparameters for the Adam optimiser are optimised,^21^ which will affect the rate at which model parameters are updated. Our MLPs all consisted of four layers: an input layer, two hidden layers and one output layer, and use the ReLU (Rectified Linear Unit) activation function to provide non‐linearity (see also Figure S2). To reduce overfitting, dropout is used after both the first and third layers. Dropout is a deep learning regularisation technique that aims to improve model generalisation and reduce overfitting, by simulating several different architectures with a single model through randomly ‘dropping out’ (removing) neurons in a network with a certain probability.^22^ The dropout probabilities in our networks, $p_{1}$ and $p_{2}$, are two additional hyperparameters that were chosen pretraining.

A description of hyperparameters for the training process and a diagrammatic representation of the MLP model is provided in Appendix S1.

The two models trained, using data from hospital 1, were then validated on data from hospital 2. We did not retrain or fine‐tune the two models using data from hospital 2. The entire dataset from hospital 2 was treated as a validation dataset, evaluating the performance of the model in a heterogenous population, thereby testing the generalisability of the model. Hospital 2 data were normalised and then passed through developed models.

We only analysed patients that had complete data needed for our analysis.

#### 2.2.4 Validation metrics

The metrics to quantitively evaluate performance of models on the test and validation datasets were as follows^23^; Area Under the Receiver Operating Characteristic curve (AUROC), a common metric used to evaluate binary classifiers. The ROC curve is a plot of the false positive rate versus true positive rate at different predictive thresholds, with AUROC being calculated as the area under this curve. This gives an idea of the predictive performance of the models and is typically used when a classifier is trained on imbalanced classes, as it is a better indicator of performance than accuracy (which will be biased if the model is always predicting the majority class). Similarly, the F1 score is defined as the harmonic mean of precision and recall: (‘precision’ × ‘recall’)/(‘precision’ + ‘recall’).

The F1 score is also used in place of simple model accuracy when the classes are imbalanced. Sensitivity describes the proportion of true positives (TP). Specificity is the proportion of true negatives (TN). In our application, a high sensitivity value was more important than specificity, as a high proportion of false negatives could compromise patient safety.

Positive predictive value (PPV) and negative predictive value (NPV) can be seen as versions of sensitivity and specificity that take disease prevalence into account.^24^ PPV (NPV respectively) is the probability that, given a positive (negative) result the patient will (not) experience deterioration. Due to the extremely low prevalence of creatinine/bilirubin deterioration, we can expect PPV to be small due to the increased number of false positives (FP)—indeed, this is further exacerbated by our model favouring FPs over false negatives (FN). False negative rate (FNR) is a simple metric defined as the overall proportion of FNs. This is a particularly useful evaluation metric for our model due to the importance of keeping the number of FNs as low as possible. Cohen's Kappa^25^ is a metric that measures the agreement between two and more judges (in this case, our models and the ground truth). It is defined as κ = (p_o‐p_e)/ (1‐p_e) where p_0 is the relative observed agreement between judges and p_e is the probability of chance agreement. Although like a simple agreement percentage calculation, Cohen's Kappa takes the probability of chance agreement into account.

Finally, to evaluate the potential clinical value of our final model, we performed a net‐benefit analysis.^26^ The most basic interpretation of the decision curve produced by a net‐benefit analysis is that the model with the highest net benefit at a particular threshold has the highest clinical value. In this analysis, three scenarios were compared: selecting all patients for the intervention (treat all, i.e. all patients receive blood tests), selecting no patients (treat none, i.e. no patients receive blood tests) and selecting patients using the predictive model. The x‐axis depicts the threshold probability, which is chosen by the decision‐maker. The y‐axis depicts the net benefit of each strategy, which is expressed in terms of the value of true positives.^26^

## 3 RESULTS

Following the defined inclusion/exclusion criteria for this study, a total of 999 (hospital 1) and 530 (hospital 2) patient records were extracted from EP systems. Some of these patients had missing data, and with such a small sample size techniques such as imputation were not appropriate, so we included only patients whose records held no missing values for any of our covariates or targets. As shown in Figure S1, this resulted in a total of 684 (hospital 1) and 530 (hospital 2) patients meeting the inclusion criteria and being included in the study.

Table 1 describes the characteristics for the populations of patients treated at the two hospitals. In total, 1214 patients were included, of which 530 were in the validation cohort. In total 184 (15%) patients experienced a one‐grade or greater change following cycle 2 of treatment; changes in creatinine were seen by 3% (*n* = 38) of the whole population, and 12% (*n* = 146) for bilirubin.

**TABLE 1:** Baseline characteristics from two hospitals.

Parameter	Hospital 1	Hospital 2
Number of patients (*N)*	684	530
Age	Median: 55; Range: (18–88)	Median: 60; Range: (18–88)
Gender	Female: 478 (70%)	Female: 337 (64%)
	Male: 206 (30%)	Male: 193 (36%)
Tumour type	Breast: 268 (39%)	Breast: 212 (40%)
	DLBCL: 182 (27%)	DLBCL: 67 (13%)
	Colorectal: 234 (34%)	Colorectal: 251 (47%)
Regimen received	FEC: 79 (12%)	FEC: 141 (27%)
	T‐FEC: 189 (28%)	T‐FEC: 71 (13%)
	RCHOP: 182 (27%)	RCHOP: 67 (13%)
	FOLFOXIRI: 1 (0.1%)	FOLFOXIRI: 0 (0%)
	IRMDG: 26 (3.5%)	IRMDG: 67 (13%)
	OXCAP: 25 (3.4%)	OXCAP: 72 (13%)
	FOLFOX: 175 (26%)	FOLFOX: 112 (21%)
Mean creatinine	65.36	69.96
Mean bilirubin	6.90	6.64
Patients with any deterioration at cycle 3 (creatinine)	27 (4%)	11 (2%)
Patients with any deterioration at cycle 3 (bilirubin)	77 (11%)	69 (13%)

Abbreviations: DLBCL, diffuse large B‐cell lymphoma; EC, epirubicin and cyclophosphamide; FEC, fluorouracil, epirubicin and cyclophosphamide; Folfoxiri, fluorouracil, irinotecan, oxaliplatin; FOLFOX, Oxaliplatin modified de gramont; IRMDG, irinotecan modified de gramont; R‐CHOP, rituximab, cyclophosphamide, doxorubicin and prednisolone.

Model performance metrics are detailed in Table 2 and shown in Figures S6 and S7 which depict the validation results. In patients where false negatives arose, we found that these grade 1 changes in creatinine and bilirubin did not result in future dose reductions, delays or omissions of dosing at cycles 3 and 4.

**TABLE 2:** Deep learning model performance mean metrics (95% CI).

	Hospital 1		Hospital 2	
Performance Metric	Creatinine	Bilirubin	Creatinine	Bilirubin
AUROC	0.99 (0.98, 1)	0.97 (0.95, 0.99)	0.76 (0.70, 0.82)	0.72 (0.68, 0.76)
F1 Score	0.99 (0.98, 1)	0.66 (0.65, 0.67)	0.59 (0.54, 0.64)	0.24 (0.14, 0.33)
Sensitivity	0.99 (0.98, 1)	0.99 (0.98, 0.99)	0.60 (0.55, 0.64)	0.54 (0.52, 0.56)
Specificity	0.99 (0.98, 1)	0.91 (0.89, 0.93)	0.98 (0.96, 0.99)	0.90 (0.87, 0.94)
PPV	0.99 (0.98, 1)	0.5 (0.5, 0.5)	0.59 (0.47, 0.71)	0.24 (0.23, 0.25)
NPV	0.99 (0.98, 1)	0.99 (0.99, 0.99)	0.99 (0.98, 1)	0.92 (0.91, 0.94)
Cohen's Kappa	0.99 (0.98, 1)	0.65 (0.60, 0.69)	0.57 (0.5, 0.64)	0.20 (0.10, 0.31)
FNR	0.01 (0, 0.02)	0.00 (0.00, 0.00)	0.31 (0.27, 0.36)	0.37 (0.28, 0.45)

Abbreviations: AUROC, area under receiver operator characteristic curve; FNR, false negative rate; NPV, negative predictive power; PPV, positive predictive power.

**FIGURE 1:**
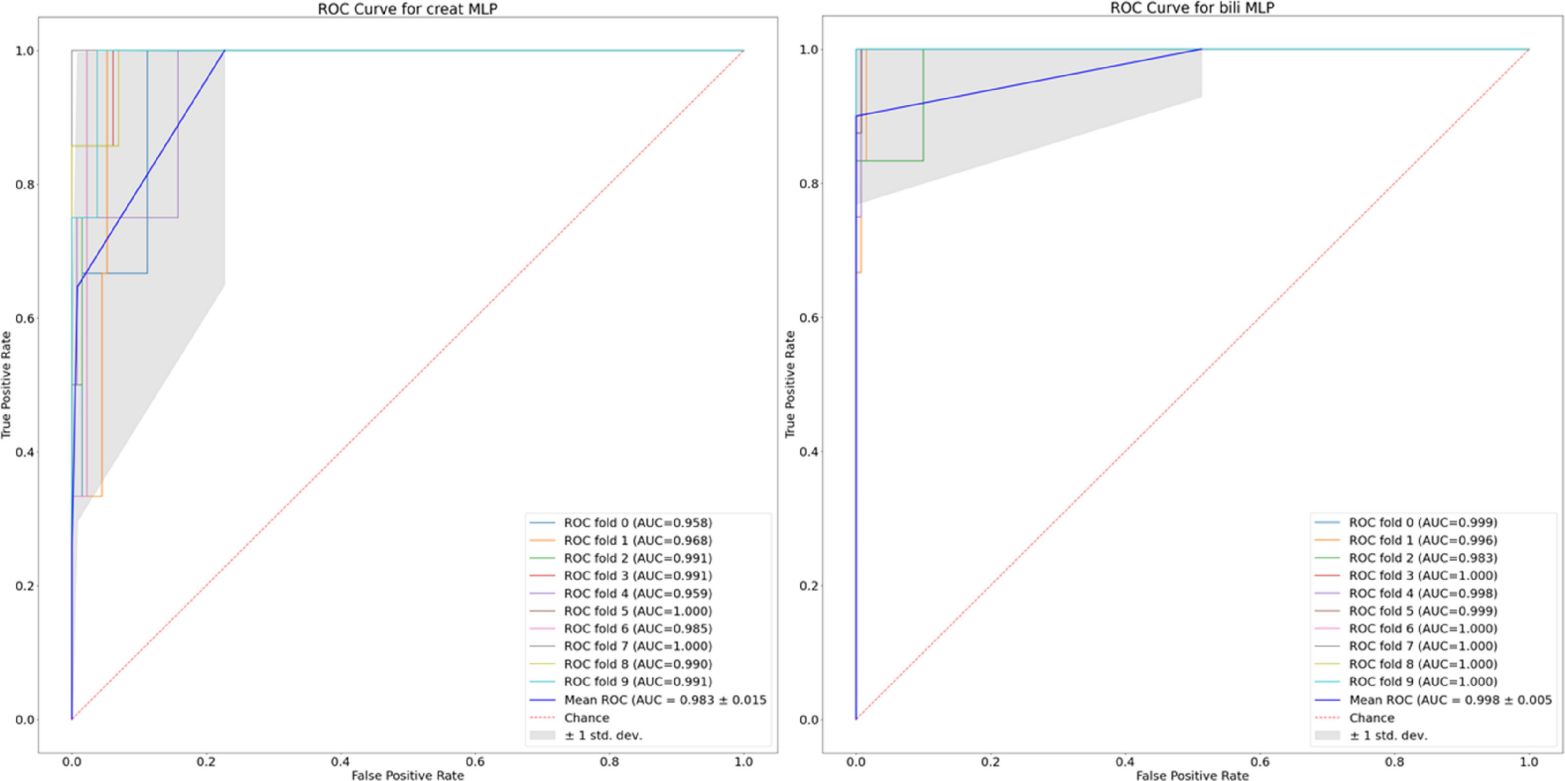
Receiver operating characteristic (ROC) curves and associated area under the ROC curves (AUROC) for each of the 10 cross validation folds on creatinine (left) and bilirubin (right). To allow for the ROC curves to be computed, the output (i.e. predicted creatinine/bilirubin values) of each model is first normalised. The true positive rate (TPR) and false positive rate (FPR) is then calculated when using different thresholds of the predicted value to classify patients as experiencing a grade change. For example, a threshold of 0.5 would result in any patient with a (normalised) predicted creatinine/bilirubin value greater than 0.5 to be predicted as undergoing a grade change. The dashed red line shows the ROC curve for a theoretical model that is no better than randomly guessing the outcome.

The Area Under Receiver Operator Characteristic curve (AUROC) showed excellent discrimination in both the training dataset (hospital 1) and the validation dataset (hospital 2). The AUROC continues to be high in validation (hospital 2), with values of 0.76 (95% CI 0.70–0.82) and 0.72 (95% CI 0.70–0.74) for creatinine and bilirubin, respectively. Figure 1 illustrates the ROC curves for both creatinine and bilirubin, across each of the 10 cross validation folds used during training on the hospital 1 training data. Differences in ethnicities were seen upon comparison of hospital 1 and 2 data, which may account for differences in model performance. Differences in ethnicities between the two datasets are shown in Figure S4. However, on undertaking a visualisation and error analysis detailed in full methodology (Appendix S1), we found no differences in model performance between the three cancer types investigated.

We note that the model performs less effectively for bilirubin than it does for creatinine. There is also a more significant drop between the two hospitals for bilirubin than there is for creatinine; this is particularly evident when inspecting the PPV and Cohen's Kappa values. However, these values are calculated using the number of true positives, which, due to the extremely small number of patients with adverse bilirubin values, are extremely sensitive to false negatives. This is a known issue with the PPV metric, which is extremely sensitive to the prevalence of the disease, and as such all evaluation metrics should be considered when evaluating model performance. NPV has remained high in both the training and validation sets due to the low volume of false negatives.

**FIGURE 2:**
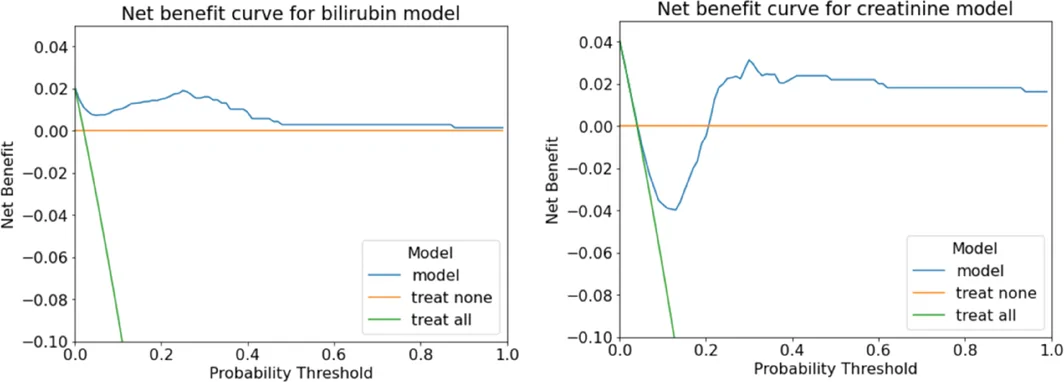
Net Benefit curves for bilirubin (left) and creatinine (right) prediction models at cycle 3. The model net benefit is calculated over the mean of all models produced during 10‐fold cross validation, as is normal procedure for ML models.^17^

Net Benefit^27^ curves for the models are shown in Figure 2. Net benefit is a method used for evaluating predictive models by exploring the clinical applicability of the model/decision approach. Decision curve analysis suggested that for the creatinine model, predicted probability cut‐offs greater than 0.2 provided greater net benefit than the competing extremes of monitoring in all patients or in none. However, at probability cut‐offs lower than 0.2, the no monitoring, or ‘treat‐none’ strategy, is superior. In the case of bilirubin, our model performs better than the extremes across all probability thresholds. This implies that both creatinine and bilirubin models added benefit to the clinical process irrespective of threshold probability used, as both our models significantly improves the current status quo of monitor all (‘treat all’ strategy).

## 4 DISCUSSION

In this study involving 1214 patients receiving chemotherapy for three different cancer types, we found the occurrence of one‐grade change in creatinine and bilirubin to be 4% and 12%, respectively. This finding strengthens our justification for the use of a model to guide stratification of these assessments of organ function in patients being treated with chemotherapy, and to reduce blood tests for most patients identified as having a low risk of organ dysfunction.

We have demonstrated the strong predictive performance of our model. Through validation using an independent dataset, we proved good performance with an AUROC of 0.76 (95% CI: 0.70, 0.82) for creatinine and 0.72 (95% CI: 0.68, 0.76) for bilirubin. Importantly, the NPV (reflecting rates of false negative results) remained high in both training and validation data.

Future application of this model in clinical practice would be dependent on the rate of false negatives. For instance, if the model predicts that there will be no deterioration in creatinine or bilirubin, but deterioration in either parameter subsequently occurs, then patient safety would in this case be compromised. In both datasets, however, the model demonstrated low false negative rates. Furthermore, all false negatives were one grade change only, and did not have any impact on subsequent cycle dosing for two further cycles, meaning there was no clinical impact on the patient.

Reducing the occurrence of false negatives was intentional, and was achieved through adjustments to the original CTCAE grade boundaries to account for small errors made by the predictive model (this is reported in Table S1). These adjustments are the reason for a lower F1 score and positive predictive values (PPV) of the models. This was also reflected in the high NPV for the data from both hospitals. False negatives could have been reduced further through penalties for missing grade changes; however, we believed this was clinically unnecessary.

When we commenced this work, we were limited by the sparsity of literature that quantified the percentage of patients that were likely to have an occurrence of the events of interest. From our data, we have now determined these values. The small proportion of patients that encounter the outcomes of interest is the biggest limitation to this work, with further validation work being required before our model can be used in clinical practice. The number of events will be used to calculate sample size for a further validation study, countering the effects of overfitting.^28^

We found that model performance reduced slightly in validation. This may be an effect of differences in ethnicity, rather than overfitting of the model. Strategies to improve the performance can be included in validation, whereby new data are simulated using the data from any underrepresented groups, fine‐tuning the model using new data for external validation. Reassuringly, we found no differences between cancer types and model performance, meaning that the model is able to adapt to differences in cancer and treatment regimens. In our future validation work we will test our model in different cancers and treatments to provide wider benefits.

Our model predicted renal and hepatic function test grade changes with great accuracy, despite lacking genetic sequencing data, cancer‐specific biomarkers or any detailed information about cancers beyond routinely collected EP data.^16^ This finding was consistent with another published model used to predict chemotherapy deaths following chemotherapy treatment.^16^ The strong performance of the model underlines the fact that common clinical data elements contained within an EP system could be used as ‘signals’ for predicting outcomes. These signals could also represent clinician behaviours. In our developed model, signals such as prescribing granulocyte colony stimulating factors and reducing cycle 1 doses for patients could indicate that the clinician felt concerned about the patient upon initiation of chemotherapy.

As all the data required to make these predictions is routinely recorded,^7^ the model could be feasibly embedded into an EP system to stratify patients into those that require monitoring for renal and hepatic function and those that do not. The model would not require manual input from clinicians, and should allow any clinician to override recommendations from the model. Our validation study is planned as prospective and across any first‐line cancer treatment, analysing patient visits saved, economic costs and the impact on the environment. In planning our next study, we have found that there is an educational need from the clinical community, to understand and trust the output of these models and to enable rapid implementation in clinical practice. We also understand the need for feature selection to allow for the proposed techniques to be applied to settings where not all required data is collected. Whilst the current number of variables used by the model is still quite small by modern machine learning standards, it would be prudent to reduce this to the minimal set needed via a variety of feature selection methods. In this work we chose not to apply such techniques due to sample size constraints for training and possible bias of features such as ethnicity, cancer type and treatment type present in this data.

There is an urgent need to use algorithmic predictions such as this, to stratify patients to manage the growing numbers of patients that will receive cancer treatment. Early identification of patients could support better safety netting for patients, whilst negating the need for testing in others. There are many other patient groups in other disease specialities that are similar to the chemotherapy population, where advances in technology can support stratification of patients to improve safety and patient experience.

In conclusion, whilst we found that the occurrence of renal and hepatic deterioration in patients receiving chemotherapy is uncommon, the opportunity exists for the incorporation of ML models into EP systems to improve the safety‐netting of some patients, and to reduce the burden of blood tests for others.

## AUTHOR CONTRIBUTIONS

**Pinkie Chambers:** Conceptualization (lead); data curation (equal); formal analysis (supporting); methodology (equal); project administration (lead); resources (lead); software (equal); supervision (equal); validation (lead); visualization (equal); writing – original draft (lead); writing – review and editing (lead). **Matthew Watson:** Conceptualization (supporting); data curation (supporting); formal analysis (lead); funding acquisition (supporting); investigation (equal); methodology (equal); project administration (supporting); resources (supporting); software (lead); supervision (supporting); validation (lead); visualization (lead); writing – original draft (supporting); writing – review and editing (supporting). **John Bridgewater:** Investigation (supporting); methodology (supporting); project administration (supporting); resources (supporting); software (supporting); supervision (supporting); validation (supporting); visualization (supporting); writing – original draft (supporting); writing – review and editing (supporting). **Martin D Forster:** Data curation (supporting); investigation (supporting); methodology (supporting); project administration (supporting); visualization (supporting); writing – original draft (supporting); writing – review and editing (supporting). **Rebecca Roylance:** Formal analysis (supporting); visualization (supporting); writing – original draft (supporting); writing – review and editing (supporting). **Rebecca Burgoyne:** Investigation (supporting); visualization (supporting); writing – original draft (supporting); writing – review and editing (supporting). **Sebastian Masento:** Investigation (supporting); methodology (supporting); visualization (supporting); writing – original draft (supporting); writing – review and editing (supporting). **Luke Steventon:** Methodology (supporting); visualization (supporting); writing – original draft (supporting); writing – review and editing (supporting). **James Harmsworth‐king:** Funding acquisition (supporting); investigation (supporting); methodology (supporting); project administration (supporting); supervision (supporting); writing – original draft (supporting); writing – review and editing (supporting). **Nick Duncan:** Data curation (equal); formal analysis (supporting); investigation (supporting); resources (supporting); supervision (supporting); validation (supporting); visualization (supporting); writing – original draft (supporting); writing – review and editing (supporting). **Noura Al‐Moubayed:** Conceptualization (equal); data curation (equal); formal analysis (equal); funding acquisition (equal); investigation (equal); methodology (lead); project administration (equal); resources (equal); software (lead); supervision (lead); validation (equal); visualization (equal); writing – original draft (equal); writing – review and editing (equal).

## CONFLICT OF INTEREST STATEMENT

NAM, JHK and MW are funded by Evergreen Life. PC reports research grants from Janssen, Pfizer, Tessaro and Bristol Myers Squibb. These grants are outside of this submitted work. MF reports research and education grants from Bristol Myers Squibb and AstraZeneca outside of the submitted work. JB, RR SM, RB, LS and ND report no conflicts of interest.

## ETHICS STATEMENT

This study was based on retrospective datasets and therefore data obtained for this retrospective study was approved by the national Review Board in addition to institutional review boards. The review board was the Health Research Authority (HRA) and approvals were required and granted on 24 November 2017 reference IRAS 226078. Information governance approvals were granted at each recruited site in accordance with hospital policies.

## Supporting information


Appendix S1

## Data Availability

The datasets generated and analysed during the current study are available from the corresponding author upon reasonable request.
