# Supplementary material for: Personalising monitoring for chemotherapy patients through predicting deterioration in renal and hepatic function
Source: Cancer Med. 2023 Aug 23;12(17):17856–65. doi: 10.1002/cam4.6418 (PMC10524043; doi:10.1002/cam4.6418)
Supplement: Supplementary file 1 — Appendix S1 [file CAM4-12-17856-s001.docx]

**Supplementary materials – Full Procedures for Model development and Validation and additional Figures.**


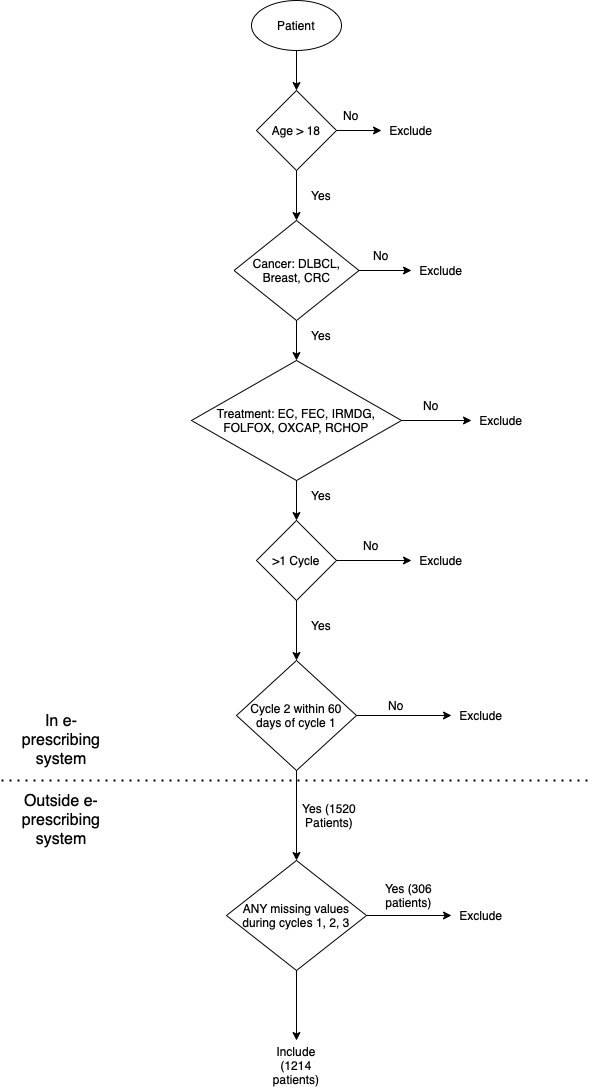


Supplementary Figure 1 – Patient flow diagram showing how patient data was selected for use in this retrospective study.

**Development model hospital 1**

Deep Learning (DL)^1^ methods were used to train two separate predictive models; one with the aim of predicting a patient’s third cycle creatinine level and the other to predict their third cycle bilirubin level. DL was chosen over traditional techniques as, on the hospital 1 validation set, MLPs were found to slightly outperform traditional techniques on the bilirubin risk prediction task. For example, whilst a trained XGBoost model achieved an F1 score of 0.99 (CI: (0.99, 1)) on the creatinine task, which is identical to that of an MLP, on the bilirubin data it achieved an F1 score of 0.65 (CI: (0.63, 0.66)). As this suggests that MLPs can slightly outperform tree-based models on this task, the rest of the study is focused solely on modern DL techniques.

MLPs are a form of deep learning that consists of an input layer (which takes raw data, i.e., the predictors used, as an input) followed by one or more fully connected ``hidden" layers. The layers are composed of individual operations (``neurons") and aim to learn ever more abstract representations of the data as it passes through the network. In this case, as our problem is a regression problem, the final output layer of the network consists of one neuron (the output of which is the final predicted value). The models were trained with the Adam optimiser, which is an extension to the classical Stochastic Gradient Descent (SGD) technique originally used to train MLPs and has been shown to have numerous benefits over SGD in many applications^2^. MLPs are trained to optimise the value of a loss function, which is chosen such that minimisation of the loss function results in maximisation of model performance. The task of predicting creatinine and bilirubin values is a regression task, and so the mean squared error (MSE) between the predicted value and true value is used as the loss function for our MLP models. An evaluation metric which quantifies the performance of the final, trained model must also be chosen. We use adjusted $R^{2}$ value as it is most appropriate for regression problems. Adjusted $R^{2}$, or $\bar{R}^{2}$ is defined as

$$\bar{R}^{2} = 1 - (1 - R^{2}) \cdot\frac{n - 1}{n - p -1}$$

where

$$R^{2}= 1 - \frac{\sum_{i} {(y_{i} - f_{i})}^{2}}{\sum_{j} {(y_{j} - \bar{y})}^{2}}$$

and $n$ is the sample size, $p$ is the total number of explanatory variables, $\bar{y}$ is the mean true value, and $y_{i}$ and $f_{i}$ are the true and predicted values for sample $i$ respectively.

Supplementary able 1 - Chosen decision boundaries used to classify a patient using their predicted third cycle creatinine/bilirubin values. ULN - Upper Limit of Normal; F – predicted value (from corresponding DL model)

|  | **Creatinine** | **Bilirubin** |
| --- | --- | --- |
| **No Grade Change** | $F\leq1.4*\text{baseline}$*;* $F\leq1.4*\text{ULN}$ | $F\leq1.4* \text{ULN}$ |
| **Grade Change** | $F>1.4* \text{baseline}$; $F>1.4*ULN$ | $F>1.4*ULN$ |

The output of the trained models is the patient's predicted creatinine/bilirubin value. These predicted values are then used to place patients into one of two groups: the patient is predicted a grade change (as defined by the CTCAE guidance), or the patient's bilirubin/creatinine grade is predicted to remain stable. These groups are defined in supplementary table 1, with the boundaries for each group being derived from CTCAE guidelines^3^. Importantly, the grade boundaries defined in this table are slightly lower than those in the original CTCAE guidance. This is by design and ensures that any patients with predicted creatinine/bilirubin values close the CTCAE grade boundaries will be classified as potentially experiencing a grade change. This accounts for any small errors in the predictive models, and although it will slightly increase the number of false positive (FP) classifications it significantly reduces the number of false negatives (FN). From a clinical perspective, a larger number of FPs is much preferable to a large number of FNs as any FP will still receive blood tests at every cycle whereas a FN patient will not.

In order to train the models on one hospital's data, the data must first be divided into training, validation, and test sets such that the models can be tested on unseen data that are not used during training; this helps avoid (and detect) the problem of overfitting. Overfit models are trained to a point where it can remember the correct targets for each individual training point used instead of learning the patterns between features and the target and so are undesirable as they will not generalise to unseen data. We use a typical 80%, 10%, 10% split for the training, validation, and test splits respectively, using stratified sampling to ensure each set is representative of the entire population. All data is first normalised to ensure that the magnitude of each feature does not grossly affect the MLPs outcome.

10-fold cross validation is used to evaluate the performance of the trained models. This technique provides a less-biased, less-optimistic evaluation of a model than just using a single train/test dataset split. To perform 10-fold cross validation, the entire dataset is split into 10 different folds. Then, for each fold, we use that as the test set and the remaining 9 groups as the training set - the results are then summarised across each of the 10 folds to gain the final performance metrics. As each sample in the dataset is part of only one of the 10 folds, each data point is used in the test set exactly once and is used to train the model exactly 9 times. As cross validation helps detect and overcome overfitting, it can be seen as an alternative to calculating the sample size needed (which we could not do due to this being a retrospective study) and estimating the statistical power of a DL classifier (which is not defined for DL models).

As part of the training process, several sets of hyperparameters are chosen - these are values that optimise the way in which the MLP models are trained. For example, the number of layers in the MLPs must be selected pre-training, as must the size of these layers. Hyperparameters for the Adam optimiser are optimised, which will affect the rate at which model parameters are updated. Our MLPs all consist of 4 layers (see also supplementary figure 2): an input layer, two hidden layers and one output layer, and use the ReLU (Rectified Linear Unit) activation function to provide non-linearity. To alleviate the risk of overfitting, dropout is used after both the first and third layers; dropout is a deep learning regularisation technique that aims to improve model generalisation and reduce overfitting by simulating several different architectures with a single model by randomly ``dropping out" (removing) neurons in a network with certain probability. The dropout probabilities in our networks, $p_{1}$and $p_{2}$ are two additional hyperparameters that are chosen pre-training.

To choose optimal values for the hyperparameters, the grid search tuning technique is used: we systematically train numerous MLPs, changing the hyperparameters each time, and use the model with the hyperparameters that produces the best results/predictions (i.e. the one with the lowest MSE loss function value and highest $\bar{R}^{2}$). The hyperparameters used in this work are reported in supplementary table 2. Throughout all experiments a batch size of 32 is used (i.e., during training the model is passed 32 training samples at a time before the model's parameters are updated) and training runs for 60 epochs (i.e. training runs through the entire training dataset 60 times).

Supplementary figure 2 is a diagrammatic representation of our MLP model followed by the binning process and can be used to visualise the flow of patient data through the system.

Supplementary table 2 - Optimal ML model hyperparameters used in final models

|  | **Model Hyperparameters** | | | | **Adam Optimiser Hyperparameters** | | |
| --- | --- | --- | --- | --- | --- | --- | --- |
| **Model** | **Layer 2 Size** | **Layer 3 Size** | **P1** | **P2** | **Learning Rate** | **Beta1** | **Beta2** |
| **Creatinine** | 120 | 100 | 0.4 | 0.3 | 0.0003426 | 0.9 | 0.999 |
| **Bilirubin** | 120 | 100 | 0.2 | 0.1 | 0.004242 | 0.9 | 0.999 |


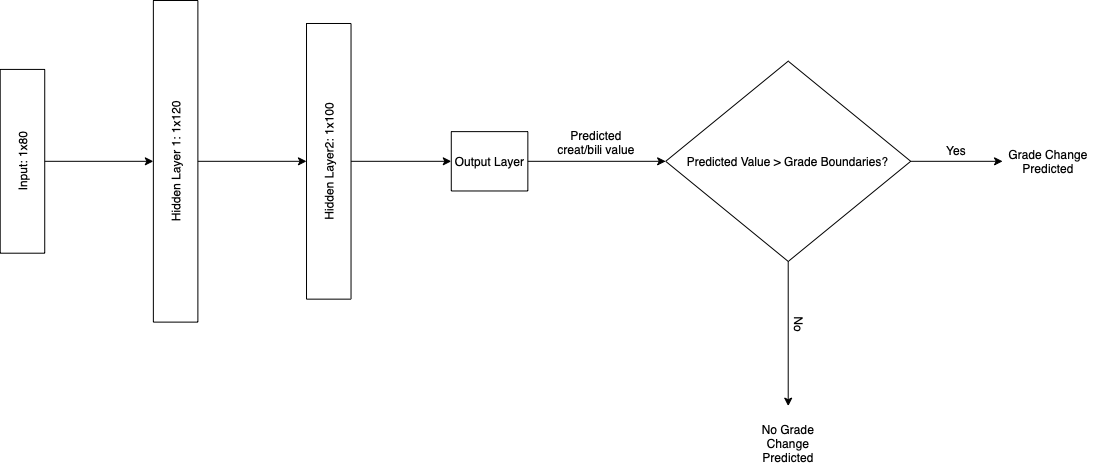


*Supplementary Figure 2. – Diagram of our MLP structure (with our chosen architecture hyperparameters) and binning process.*

Model Training/Testing Loss Curves

Comparison of the training and testing loss during model training gives insight into whether the model is overfitting or not; models that have overfit to the training data show significantly higher testing loss than training loss^4^. Supplementary figure 3 shows the mean training and testing loss across all 10 cross validation folds of training on hospital 1 data, and from this figure we can verify that the models are not overfitting.


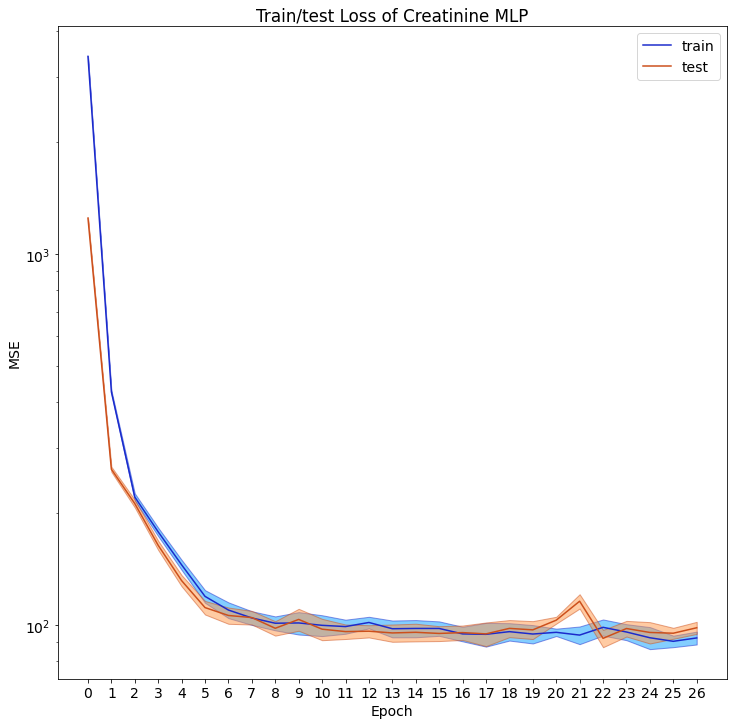

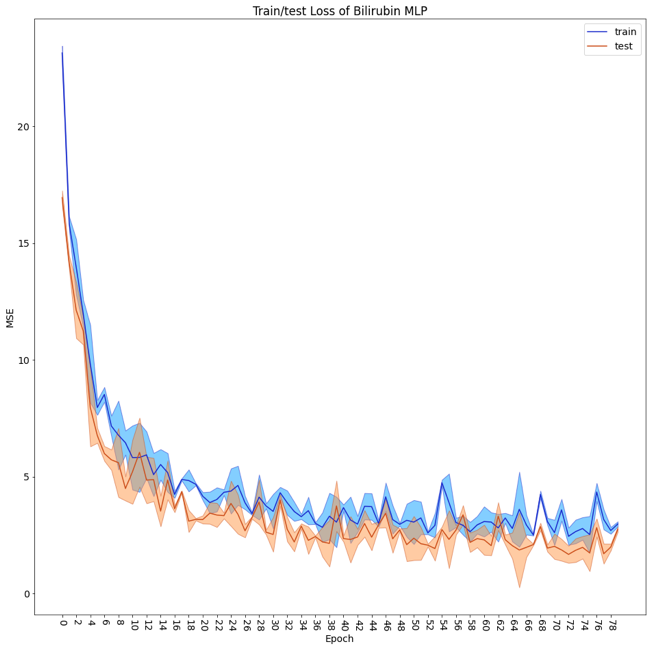


*Figure 3 –Training/Test Loss curves for the creatinine (left) and bilirubin (right) models. Each plotted line is the mean loss across all 10 folds, ± 1 standard deviation (shaded). As the training loss is always higher (or similar to) the test loss for both the creatinine and bilirubin models, we can conclude they are unlikely to be overfitting to the training data.*

#### Validation hospital Validation using hospital 2 data:

The models trained on data from hospital 1 are validated on data from a second, distinct hospital. We do not retrain, nor finetune, the two models on data from hospital 2. The entire dataset from hospital 2 is treat as a validation dataset, evaluating how well the model performs on unseen data with a different underlying population. Not only is this important from a machine learning perspective (as it ensures that the model has not been overfit to hospital 1 data) but it also validates our models' ability to generalise to data from different hospitals with different patient demographics. This is imperative for the models to be used in practice.

Hospital 2 data is first normalised, and then passed to the two models. The models then predict cycle 3 creatinine and bilirubin values, with these predicted values being used to classify the patient as experiencing/not experiencing a grade change based on the rules in supplementary table 1.

Validation Metrics

We utilise several different metrics to quantitively evaluate the performance of our models on the test and validation datasets, based on metrics used in other applications of DL to clinical practice. The Area Under the Receiver Operating Characteristic curve (AUROC) is a common metric used to evaluate binary classifiers. The ROC curve is a plot of the false positive rate vs. true positive rate at different predictive thresholds, with AUROC being calculated as the area under this curve. This gives an idea of the predictive performance of the models and is typically used when a classifier is trained on imbalanced classes as it is a better indicator of performance than accuracy (which will be biased if the model is always predicting the majority class).

Similarly, the F1 score is defined as the harmonic mean of precision and recall:

$$\frac{\text{precision}\times\text{recall}}{\text{precision}+\text{recall}}$$

The F1 score is also used in place of simple model accuracy when the classes are imbalanced. Sensitivity is the proportion of people who test positive that go on to experience creatinine/bilirubin deterioration– i.e., it is the proportion of True Positives (TP). On the other hand, specificity is the proportion of people who test negative that do not go on to experience deterioration – i.e., the proportion of True Negatives (TN). In our application, a high sensitivity value is more important than specificity – it is important that patients classified as negative by our model do not go on to experience any deterioration.

Positive Predictive Value (PPV) and Negative Predictive Value (NPV) can be seen as versions of sensitivity and specificity that take disease prevalence into account; PPV (NPV respectively) is the probability that, given a positive (negative) result the patient will (not) experience deterioration. Due to the extremely low prevalence of creatinine/bilirubin deterioration, we can expect PPV to be small due to the increased number of False Positives (FP) – indeed, this is further exacerbated by our model favouring FPs over False Negatives (FN). False Negative Rate (FNR) is a simple metric defined as the overall proportion of FNs. This is a particularly useful evaluation metric for our model due to the importance of keeping the number of FNs as low as possible.

Cohen’s Kappa is a metric that measures the agreement between two or more judges (in this case, our models and the ground truth). It is defined as

$$\kappa=\frac{p_{o}-p_{e}}{1-p_{e}}$$

where $p_{0}$is the relative observed agreement between judges and $p_{e}$is the probability of chance agreement. Although similar to a simple agreement percentage calculation, Cohen’s Kappa takes the probability of chance agreement into account.

Dataset Visualisation and Error Analysis

The reduction in performance of the models when applied to the hospital 2 data is to be expected – the patient population will be different to that the models was trained on. Indeed, this difference can be visualised by plotting the first two principal components after performing Principal Component Analysis (PCA) in supplementary figure 5, where there is a clear boundary between the two hospitals. One can also see that there are clear differences in the patient population by inspecting the dataset statistics reported in Table 1 and in supplementary figure 4 – for example, the distribution of ethnicities is significantly different. Specifically, these differences in patient population are exactly why this hospital was chosen for model validation - it shows that the model is still able to perform comparatively well even with significant data shift.


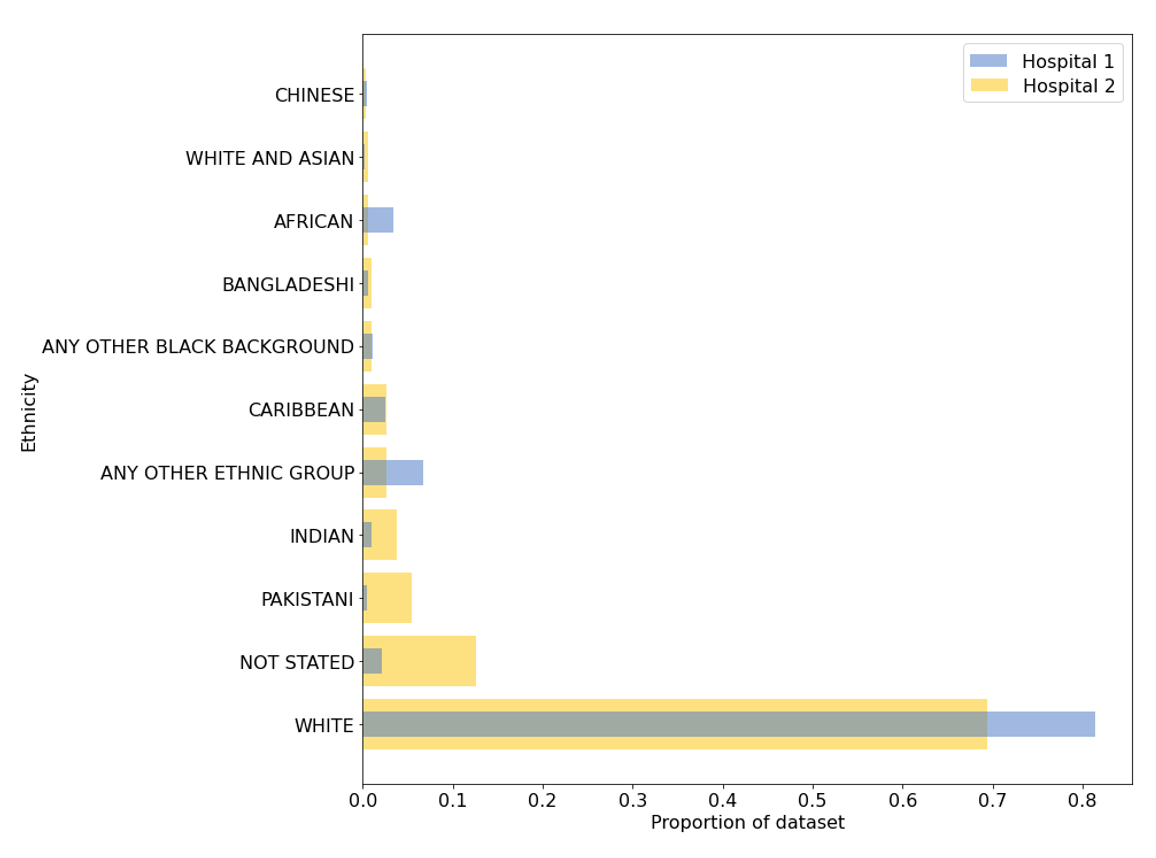


Supplementary Figure 4 - Histogram highlighting differences in patient demographics between the two hospitals. Blue lines highlight patient distribution in Hospital 1. Yellow shows the distribution in Hospital 2. As the data is from two different hospitals, they will necessarily have different patient distributions.


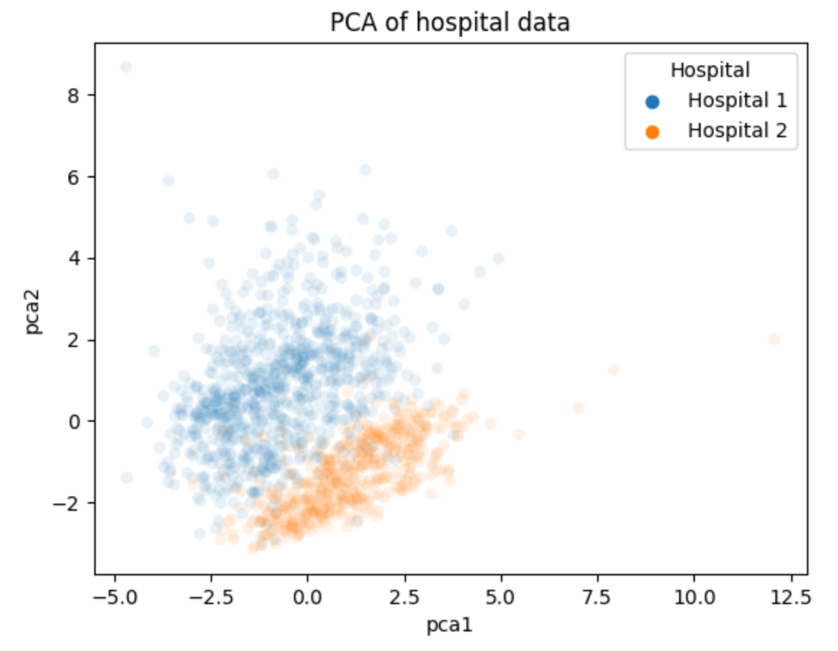


Supplementary Figure 5*– Scatterplot of the first two principal components from a Principal Component Analysis of the two datasets used for model validation and training.*

Furthermore, it is not the case that our model outperforms on one cancer type to the detriment of the others. Supplementary table 2, which is a breakdown of model performance by cancer type on hospital 2 validation data, shows that neither the creatinine nor the bilirubin model significantly outperforms on any of the three cancer types present in the dataset. This is a significant result, as it highlights that the model is able to adapt to differences in cancer and treatment regimens and suggests that any future studies that include a more varied dataset may also gain promising results.

Supplementary table 2– F1 score of the MLP models on hospital 2 validation data, stratified by cancer type.

| **Cancer Type** | **Creatinine** | **Bilirubin** |
| --- | --- | --- |
| Breast | 0.56 | 0.22 |
| DLBCL | 0.60 | 0.24 |
| Colorectal | 0.58 | 0.26 |


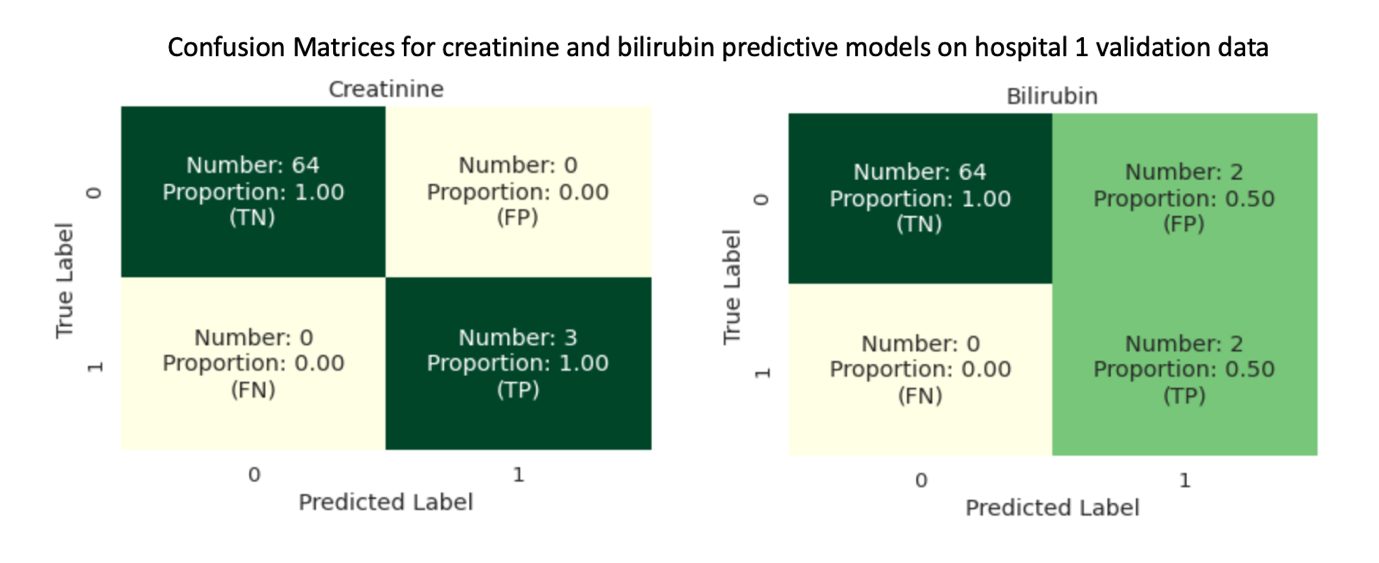


Supplementary figure 6- Confusion matrices showing the number of true negatives (TN), false positives (FP), false negatives (FN) and true positives (TP) of the best performing predictive models on hospital 1 validation data. Due to imbalanced classes, the data has been normalised across columns – this is shown as “Proportion” in each cell.


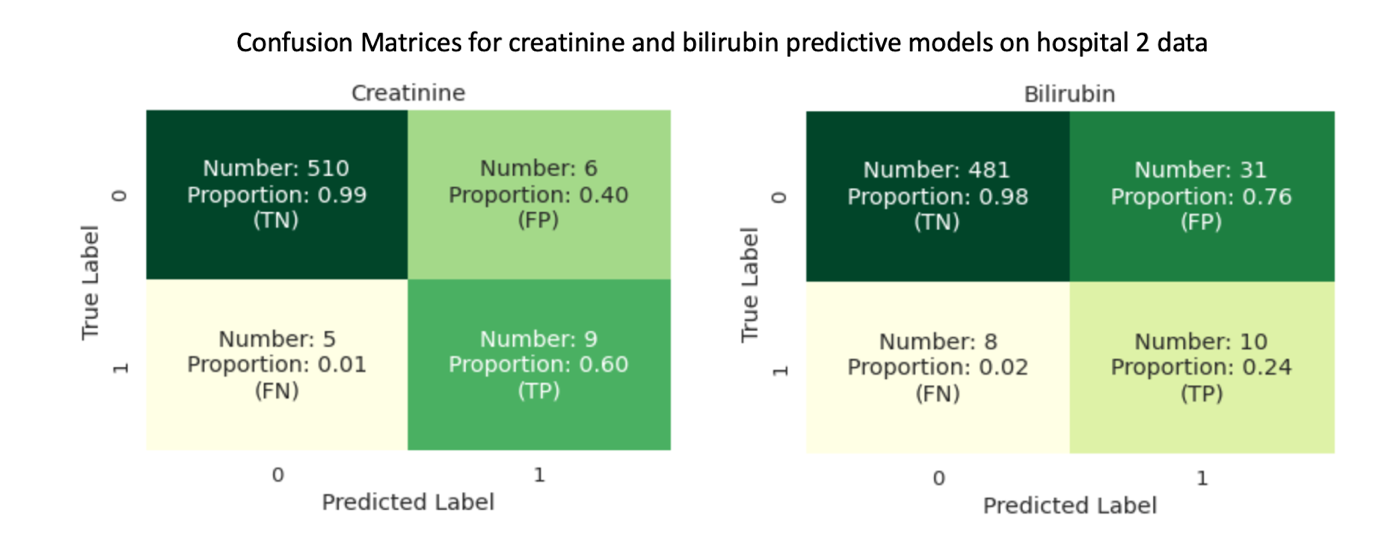


Supplementary figure 7 - Confusion matrices showing the number of true negatives (TN), false positives (FP), false negatives (FN) and true positives (TP) of the best performing predictive models on hospital 2 data. Due to imbalanced classes, the data has been normalised across columns – this is shown as “Proportion” in each cell.

# Bibliography

| [1] | A. Esteva, A. Robicquet, B. Ramsundar, V. Kuleshov, M. DePristo, K. Chou, C. Cui, G. Corrado, S. Thrun and J. Dean, "A guide to deep learning in healthcare," *Nature Medicine,* pp. 24-29, 2019. |
| --- | --- |
| [2] | D. P. Kingma and J. Ba, "Adam: {A} Method for Stochastic Optimization," in *International Conference on Learning Representations (ICLR)*, 2015. |
| [3] | National Cancer Institute, "Common Terminology Criteria for Adverse Events," [Online]. Available: https://ctep.cancer.gov/protocoldevelopment/electronic_applications/ctc.htm. [Accessed 07 03 2022]. |
| [4] | X. Ying, "An Overview of Overfitting and its Solutions," *Journal of Physics: Conference Series,* vol. 1168, 2019. |
